# Deep Brain Stimulation Can Preserve Working Status in Parkinson's Disease

**DOI:** 10.1155/2015/936865

**Published:** 2015-07-30

**Authors:** Gabriella Deli, István Balás, Tamás Dóczi, József Janszky, Kázmér Karádi, Zsuzsanna Aschermann, Ferenc Nagy, Attila Makkos, Márton Kovács, Edit Bosnyák, Norbert Kovács, Sámuel Komoly

**Affiliations:** ^1^Department of Neurology, University of Pécs, Rét Utca 2, Pécs 7623, Hungary; ^2^Department of Neurosurgery, University of Pécs, Rét Utca 2, Pécs 7623, Hungary; ^3^MTA-PTE Clinical Neuroscience MR Research Group, Rét Utca 2, Pécs 7623, Hungary; ^4^Department of Neurology, Kaposi Mór County Hospital, Tallián Gyula Utca 16, Kaposvár 7400, Hungary

## Abstract

*Objectives*. Our investigation aimed at evaluating if bilateral subthalamic deep brain stimulation (DBS) could preserve working capability in Parkinson's disease (PD).* Materials*. We reviewed the data of 40 young (<60 year-old) PD patients who underwent DBS implantation and had at least 2 years of follow-up. Patients were categorized based on their working capability at time of surgery: “active job” group (*n* = 20) and “no job” group (*n* = 20). Baseline characteristics were comparable. Quality of life (EQ-5D) and presence of active job were evaluated preoperatively and 2 years postoperatively.* Results*. Although similar (approximately 50%) improvement was achieved in the severity of motor and major nonmotor symptoms in both groups, the postoperative quality of life was significantly better in the “active job” group (0.687 versus 0.587, medians, *p* < 0.05). Majority (80%) of “active job” group members were able to preserve their job 2 years after the operation. However, only a minimal portion (5%) of the “no job” group members was able to return to the world of active employees (*p* < 0.01).* Conclusions*. Although our study has several limitations, our results suggest that in patients with active job the appropriately “early” usage of DBS might help preserve working capability and gain higher improvement in quality of life.

## 1. Introduction

The 27-year-old deep brain stimulation (DBS) revolutionized the treatment of movement disorders including drug-resistant tremor, advanced Parkinson's disease (PD) [1, 2], and dystonia [3]. Based on its high efficacy and relatively small side effect profile, more than 100,000 patients have undergone DBS implantation worldwide [4]. Approximately 80% of indications for DBS are the pharmacologically not efficiently treatable PD and considerably less patients receive DBS for other movement disorders [3, 5]. The most frequently applied surgical target for PD is the bilateral subthalamic DBS (STN DBS) capable of improving all cardinal symptoms. Besides the symptomatic improvement, STN DBS can also dramatically and permanently extend the ON time [6, 7] and the health-related quality of life (HRQoL) [8, 9].

According to the current guidelines, STN DBS is only indicated in the cases of drug-resistant tremor or severe motor fluctuations unmanageable by pharmacological treatment. The average disease duration at the time of surgery is around 15 years [5], by when the health-related quality of life (HRQoL) and sociocultural functioning are usually impaired [10]. In general, the longer disease duration is associated with the more likely appearance of levodopa-resistant symptoms and therefore DBS-resistant symptoms and higher impact on the working capability. One of the most important parts of patient selection therefore is the appropriate timing of surgery [1, 11]. If the DBS implantation is preformed “too late,” the presence and severity of DBS-resistant symptoms (e.g., postural instability, neurocognitive impairment, or speech problems) might interfere with or worsen the outcome. On the contrary, if the surgery is performed “too early,” we might operate on those patients who could have been otherwise well treated pharmacologically and needlessly expose them to the potential surgical risks. Moreover, with “too early” operations we might also include some nonidiopathic cases because the atypical features might be hidden in the early stages of the disease course.

Based on the hypothesis [12–14] that the STN DBS treatment applied at earlier stages of the disease may be superior to the best medication, a multicenter study, called EarlyStim, was initiated [15]. In this prospective study, patients receiving STN DBS had significantly larger improvement in HRQoL (−7.8 improvement on PDQ-38) than patients on best medical treatment (+0.2 points worsening, *p* = 0.002) [15]. Although the contributors of EarlyStim study state that, in well-selected cases where the “early” fluctuations impair the sociocultural functioning and HRQoL, STN DBS might be superior to optimal pharmacological treatment [15–17], there are lots of debates on this issue [18, 19].

Inspired by the results of EarlyStim study, our research group tried to evaluate if STN DBS might have an impact on the working status of PD patients. Our a priori hypothesis was that STN DBS could preserve working capability of patients having an active job at the time of DBS implantation.

## 2. Materials and Methods

### 2.1. Patients

Those patients who were included in the present study underwent bilateral STN DBS implantation at University of Pécs and participated in our prospective DBS registry. All patients signed an informed consent form approved by the Regional Ethical Board of University of Pécs. Patients were eligible for STN DBS surgery (and therefore for participating in our DBS registry) if they had the clinical diagnosis of PD in accordance with the UK Brain Bank criteria [20] and at least 5 years of documented disease duration, were under the age of 75 years, had Parkinsonian motor symptoms or dyskinesia that limited their ability to perform the activities of daily living despite optimal oral pharmacological treatment, had no dementia or major psychiatric illness, and had no contraindication to surgery. Presence of dementia was evaluated by the Hungarian validated version of Mattis Dementia Rating Scale (MDRS) [21]. The scores on MDRS can range from 0 to 144 with lower values indicating more severe dementia. Scores on MDRS ≤125 points [21] and/or fulfillment of Diagnostic and Statistical Manual of Mental Disorders 4th Edition Text Revision criteria for dementia were the exclusion criteria for STN DBS surgery.

Out of the group of patients having at least 2 years of postoperative follow-up, first we identified those patients who had an active job at the time of their STN DBS surgery and whose age was comparable with the inclusion criteria of EarlyStim study (18–60 years) [17]. Having an active job was assessed by direct inquiry. Only regular (>1 day/week), either part-time or full-time, work was defined as active job. Working capability was categorized into the following groups:

- Full-time work (regular work, 5 days/week and 8 hours/day).
- Part-time work (regular work, 1–5 days/week, 4–8 hours/day).
- Not working, retired due to the disease.
- Not working, retired not due to the disease.
- Never worked.

However, we did not consider those who participated only in housework or performed hobby activities or unpaid and irregular tasks as active workers. Altogether 20 PD patients were identified meeting the above mentioned criteria whom we classified into the group of “active job.”

To perform pairwise comparison, we chose another 20 patients out of our registry who did not have an active job at the time of their surgery (“no job” group) by the utilization of a custom-made program. The automatic selection process was made in a way that for each participant in the “active job” group we picked a “partner” who had similar age, disease duration, and fluctuation duration (in the range of ±2 years) and the same disease type (tremor-dominant versus rigid-akinetic type). These matched patients were considered as the “no job” group. We utilized this automatic pairwise selection process to create a “no job” group with balanced and comparable baseline characteristics to the “active job” group.

### 2.2. Applied Tests

Changes in the working capability and the health-related quality of life were considered as coprimary endpoints. Our primary aim was to identify what portion of young patients having active job at the time of DBS surgery maintained their active job 2 years postoperatively. On the contrary, we also investigated how many young patients not having an active job at DBS initiation returned to work.

For evaluating HRQoL, the EuroQol Instrument (EQ-5D) was assessed. Because the usage of EQ-5D requires only 2 minutes and it was available in validated Hungarian version [22] at the start of our DBS registry project, we chose this HRQoL scale (and not the PDQ-39). EQ-5D had been previously validated [23–25] and utilized in the evaluation of different therapeutic approaches in PD [26, 27]. Moreover, it can also be applied to health-related economical calculations [28]. EQ-5D consists of two major parts: a five-item questionnaire and a visual analogue scale (VAS). The first part of EQ-5D maps five different domains of HRQoL: mobility, self-care, usual activities, pain/discomfort, and anxiety/depression [22]. Based on the responses for the five domains questionnaire, an index value vas calculated (coprimary endpoint). The EQ-5D index can be in the range from −0.52 to +1, the former representing a state worse than death and the latter representing the best health-related status [22]. For the Hungarian population, a change larger than 0.0705 denotes clinically meaningful difference [29]. The response on VAS can range from 0 to 100, the higher values meaning better HRQoL [22].

Changes in major motor and nonmotor symptoms were considered as secondary endpoints of the study. Severity of Parkinson's disease was rated by both Hoehn-Yahr Scale (HYS) and Unified Parkinson's Disease Rating Scale [30]. In agreement with the recommendations of the Movement Disorders Society Task Force [31], the original (and not the modified) HYS was utilized. Therefore, in our study, the stage of 2.5 according to the modified HYS was considered as stage 3 (original HYS). The most important secondary outcome of our study was the UPDRS Part III (Motor Examination), where the score can range between 0 and 108 with higher scores indicating worse function [30]. The secondary outcome measures also included changes in activities in daily living measured by the UPDRS Part II and Schwab and England Scale (SES) [32]. Scores for the UPDRS-II can range from 0 to 52 points with higher scores indicating worse function [30]. The scores for SES can be in the range of 0–100 with higher values indicating better function. For neuropsychiatric outcomes, the MDRS and Montgomery-Asberg Depression Rating Scale (MADRS) were assessed. Scores for MADRS can range from 0 to 60, with higher values indicating more severe depression.

Each scale was assessed by three times (baseline, 1 week preoperatively, and follow-ups, 12 and 24 months postoperatively). All sessions were videotaped enabling us to reevaluate the HYS and UPDRS Part III with the exception of rigidity by a blinded rater. Amount of antiparkinson medication was calculated in levodopa equivalent dosage (LED) [33].

### 2.3. Statistics

All statistical measurements were performed by the IBM SPSS software package (IBM Inc., USA, version 22.0.1). The level of statistical significance was set at 0.05. Because most parameters did not follow the normal distribution, nonparametric tests were utilized and median values with interquartile range (IQR: 25th–75th percentile) were calculated.

Changes within each group (baseline versus follow-ups) were tested by Friedman test (baseline versus 1st year of follow-up versus 2nd year of follow-up). For intergroup analyses (e.g., “active job” group versus “no job” group) Mann-Whitney tests were applied. To evaluate changes in dichotomous variables (e.g., having or not having an active job), McNemar test was used.

To overcome the limitations of multiple comparisons, we also applied a mixed-model two-way ANOVA where the first factor has 2 levels and is independent (2 groups: having an active job and not having a job) and the second factor has 3 levels and is repeated (baseline, 1 year, and 2 years). Because ANOVA can provide the difference between the 2 groups at all endpoints, there is no need for further post hoc analyses. Furthermore, using this design, we can also assess the interactions. Because simulation studies using a variety of nonnormal distributions have shown that the false positive rate is not affected very much by this violation of the normality assumption [34–36], the nonnormal distribution of the data did not preclude using such a statistical design.

## 3. Results

### 3.1. Study Population

For the final analyses, the data of only 20 pairs were included. Due to the pairwise group selection, the most important baseline PD characteristics were comparable (e.g., age, sex, disease duration, disease type, and HYS, Table 1). Although we could not identify any significant differences, the HY staging favored the “no job” group by having more Stage 2 patients than the “active job” group did.

The dosage of antiparkinson medication, severity of motor symptoms (UPDRS-III), major neuropsychiatric symptoms (MADRS and MDRS), and HRQoL (EQ-5D index and VAS) were also similar at baseline (Table 2).

### 3.2. Working Capability

At baseline, 18 patients had a full-time job and two patients had a part-time job in the “active group.” Two years postoperatively, 16 patients from the “active job” group (80%) still had an active job (full-time job: 8 patients; part-time job: 8 patients). The reasons for work discontinuation included the reach of official age limit for pension (*n* = 1) and PD-related problems interfering with working capability (e.g., fatigue and some degree of fluctuation, *n* = 3).

Despite the comparable baseline characteristics and similar improvements in the motor symptoms and activities of daily living, only a single person (5%) from the “no job” group returned to the world of active work (McNemar test; *p* < 0.01).

### 3.3. HRQoL

Both groups had similar HRQoL at baseline (EQ-5D index values: 0.477 and 0.429, median values). These values were below the 25th percentile of Hungarian population norms [22]. (The 25th percentile population norms for the 45–54 and 55–64 years age groups are 0.69 and 0.62, resp.) After bilateral STN DBS implantation, the EQ-5D index significantly improved in both groups (Table 2, Friedman tests), which clearly exceeded the threshold of minimal clinically important difference (0.0705) [29]. However, 2 years after the operation, the “active job” group members had significantly better HRQoL than the “no job” patients did (Mann-Whitney test, *p* < 0.001, Table 2) and this difference was also clinically meaningful. Therefore, the between-groups comparisons revealed better improvement in the primary outcome (HRQoL) in the “active job” group. The application of mixed-model two-way ANOVA with Bonferroni correction further supported that HRQoL 2 years postoperatively was better in the “active job” group than in the “no job” group.

### 3.4. Secondary Outcomes

As far as the motor symptoms were concerned (UPDRS-III), both groups had similar baseline characteristics and experienced similar improvement after DBS implantation. Two years after the surgery, the motor severity was still comparable in both groups. Moreover, the changes in activities of daily living (SES and UPDRS-II) and antiparkinson medication were also similar in both groups. The only difference in the secondary outcomes was the significant improvement in MADRS score revealed by Friedman test, which was present in the “active job” group but was missing in the “no job” group. The application of mixed-model two-way ANOVA with Bonferroni correction did not identify any differences in the secondary outcomes.

## 4. Discussion

Our primary aim was to evaluate the hypothesized effect of STN DBS on preserving the working capability of PD patients. In our study, only those patients who were young (<60 years) and had at least 2-year follow-up were included. In the “active job” group, the participants had an active job at the time of surgery but their working capability was impaired by the motor symptoms (both tremor and fluctuations) to some extent. For the “no job” group, we selected patients having similar demographic and PD-related baseline characteristics to perform reliable between-group comparisons.

Our aim was to compare the efficacy of STN DBS on patients having an active job (“active job” group) at the time of surgery to the efficacy of STN DBS on patients without an active job (“no job” group). In case of tremor-dominant patients, the presence of drug-resistant tremor was the indication for surgery, whereas, in rigid-akinetic patients, the presence of severe fluctuations was the indication for surgery. Because in both groups the number of tremor-dominant and rigid-akinetic patients was identical due to the pairwise selection, we believe our study design was suitable to draw conclusions.

In the present study, the main focus was to reveal if having an active work at the time of DBS implantation could be a prognostic factor for outcome and this working capability could be preserved by STN DBS. One of the most important findings of our study was that 80% of patients having an active job at the time of surgery still had an active job 2 years after the DBS implantation. Nevertheless, only a single patient returned to the world of work in the “no job” group after the successful STN DBS therapy. Therefore, we can conclude that DBS might help preserve the working capability if it is performed in patients with active job. On the contrary, if DBS implantation is scheduled after losing the working capability, it might be insufficient to help patients return to work.

The coprimary outcome variable was the change in HRQoL. Patients in the “active job” group experienced higher improvement in HRQoL than patients in the “no job” group did despite the similar changes in motor and major nonmotor symptoms. This finding might suggest that having an active job at the time of DBS surgery might have a beneficial effect on the long-term outcome by being a positive predictive factor.

The only difference in the secondary outcomes was the significant improvement in MADRS score revealed by Friedman test. Because it was not confirmed by the multivariate ANOVA, we considered this difference both clinically and statistically irrelevant.

The authors are aware of the major limitations of their study: not being randomized, placebo-controlled, double-blind, multicenter, and prospective and having a relatively small sample size. However, our results nicely fit to the concept of EarlyStim because in some individuals the application of “early” DBS might have a beneficial role in the sociocultural functioning. According to our results, in patients with active job, the appropriately “early” usage of STN DBS might help preserve sociocultural functioning and the working capability in a two-year time frame and gain higher improvement in HRQoL. Despite similar symptomatic control, patients receiving STN DBS after losing their working capability seldom return to work again. In the opinion of the authors, having an active job at the time of surgery might be a positive predicting factor for a good outcome. Because the maintenance of working capability is beneficial not only for the patients but also for the healthcare providers, further, larger, controlled trials are warranted to confirm this hypothesis.

## Figures and Tables

**Table 1:** The demographic and disease-specific characteristics of the study population at baseline examination.

	Active job group (at the time of surgery)					No job group (at the time of surgery)					Statistics
	Median	Percentile 25	Percentile 75	Mean	Standard deviation	Median	Percentile 25	Percentile 75	Mean	Standard deviation	
Age	53	50	56	52.6	4.4	53	50	57	53.1	4.3	0.735

Sex	15M/5F					15M/5F					NA

Education level, y	12	11	13	11.9	1.6	12	11	13	11.8	1.7	0.879

Disease duration, y	8	7	10	8.2	1.8	8	7	10	8.2	1.6	0.934

Levodopa usage, y	6	5	8	6.8	2.1	7	6	8	6.9	1.5	0.619

Fluctuation, y	4	3	6	4.8	2.1	5	4	6	4.9	1.5	0.619

PD type (tremor/rigid-akinetic)	9T/11RA					9T/11RA					NA

HYS-1	0					0					0.519
HYS-2	7					9					
HYS-3	13					11					

All statistical analyses were performed by Mann-Whitney test with the exception of HYS, where Chi-square test was utilized.

HYS = Hoehn-Yahr Stages; NA = not applicable; PD = Parkinson's disease; M = males; F = females, y = years.

**Table 2:** Comparison of “active job” and “no job” groups regarding the achieved improvements in health-related quality of life and major symptoms of Parkinson's disease after bilateral subthalamic deep brain stimulation.

		Active job group (at the time of surgery)						No job group (at the time of surgery)						Between groups (Mann-Whitney)
		Median	Percentile 25	Percentile 75	Mean	Standard deviation	Friedman test (within group)	Median	Percentile 25	Percentile 75	Mean	Standard deviation	Friedman test (within group)	
EQ-5D	Baseline	0.477	0.116	0.605	0.391	0.307		0.429	0.255	0.666	0.411	0.308		0.779
	1 year	**0.660**	**0.530**	**0.770**	**0.661**	**0.163**	*<0.001 *	**0.507**	**0.439**	**0.691**	**0.543**	**0.183**	*<0.001 *	**0.035**
	2 years	**0.687**	**0.620**	**0.811**	**0.710**	**0.138**		**0.587**	**0.482**	**0.742**	**0.606**	**0.191**		**0.045**

EQ-5D VAS	Baseline	70	55	80	68.1	14.0		67	50	79	64.4	17.7		0.495
	1 year	**84**	**71**	**93**	**80.4**	**14.9**	*<0.001 *	**70**	**60**	**82**	**69.4**	**16.2**	*<0.001 *	**0.037**
	2 years	**88**	**77**	**90**	**81.2**	**15.0**		**73**	**60**	**80**	**71.5**	**14.9**		**0.021**

SES	Baseline	70	60	80	71.5	11.8		70	65	80	71.0	15.5		0.845
	1 year	80	75	90	82.0	11.5	*<0.001 *	80	70	80	76.5	11.8	*0.002 *	0.153
	2 years	**80**	**80**	**90**	**81.5**	**11.4**		**70**	**59**	**80**	**68.9**	**11.9**		**0.001**

UPDRS-III	Baseline	25	22	29	25.6	6.3		24	19	30	23.9	7.2		0.506
	1 year	22	18	26	22.1	5.3	*<0.001 *	22	18	27	22.0	6.3	*<0.001 *	0.839
	2 years	22	17	24	20.8	5.0		20	17	26	20.8	6.4		0.860

UPDRS-II	Baseline	15	14	19	15.4	4.7		15	12	19	14.9	5.3		0.724
	1 year	13	10	17	12.9	4.3	*<0.001 *	16	10	19	14.8	6.3	*<0.001 *	0.243
	2 years	12	9	16	12.2	4.9		15	11	19	15.1	6.9		0.116

MADRS	Baseline	7	6	10	7.9	3.8		8	5	9	7.9	3.2		0.744
	1 year	6	5	9	6.8	2.6	*0.020 *	7	5	10	7.4	3.5	*0.590 *	0.395
	2 years	5	5	7	5.7	1.6		7	5	10	7.4	3.2		0.066

MDRS	Baseline	135	130	139	134.5	5.6		136	130	143	136.1	6.2		0.342
	1 year	134	128	136	133.1	5.8	*0.246 *	133	127	140	133.1	6.2	*0.215 *	0.903
	2 years	134	130	137	132.7	6.3		133	127	140	133.2	6.2		0.839

LED	Baseline	905.0	780.0	1060.0	972.9	379.2		950	750	1000	925.0	265.3		0.903
	1 year	475.0	325.0	700.0	535.1	315.0	*<0.001 *	509.0	400.0	678.5	559.7	216.6	*<0.001 *	0.447
	2 years	500.0	375.0	700.0	552.5	250.5		525.0	406.0	700.0	575.0	186.5		0.463

Friedman test was used to evaluate within-group changes (comparison of baseline values with 1st year and 2nd year of follow-up). Mann-Whitney test was applied to detect between-groups differences (e.g., comparison of “active job” group versus “no job” group).

HYS = Hoehn-Yahr Stages; LED = levodopa equivalent dosage; MADRS = Montgomery-Asberg Depression Rating Scale; MDRS = Mattis Dementia Rating Scale; SES = Schwab and England Scale; UPDRS-II = Activities of Daily Living (Part II of UPDRS); UPDRS = Unified Parkinson's Disease Rating Scale; UPDRS-III = Motor Examination (Part III of UPDRS); EQ-5D = EuroQol Instrument.

## Abbreviations

DBS: Deep brain stimulation
EQ-5D: EuroQol Instrument
HRQoL: Health-related quality of life
HYS: Hoehn-Yahr Stage
LED: Levodopa equivalent dosage
MADRS: Montgomery-Asberg Depression Rating Scale
MDRS: Mattis Dementia Rating Scale
PD: Parkinson's disease
SES: Schwab and England Scale
STN DBS: Bilateral subthalamic deep brain stimulation
UPDRS: Unified Parkinson's Disease Rating Scale
VAS: Visual analogue scale (included in EQ-5D).
